# Systemically administered allogeneic mesenchymal stem cells do not aggravate the progression of precancerous lesions: a new biosafety insight

**DOI:** 10.1186/s13287-018-0878-1

**Published:** 2018-05-11

**Authors:** Flavia Bruna, Anita Plaza, Martha Arango, Iris Espinoza, Paulette Conget

**Affiliations:** 1 Centro de Medicina Regenerativa, ICIM, Facultad de Medicina Clinica Alemana Universidad del Desarrollo, Av. Las Condes 12,438, Lo Barnechea, Santiago, Chile; 2 Present Address: Laboratorio de Hormonas y Biologia del Cancer, Instituto de Medicina y Biologia Experimental de Cuyo (IMBECU), Mendoza, Argentina; 3 Present Address: Unidad de Nefrologia, Instituto de Medicina, Facultad de Medicina, Universidad Austral de Chile, Valdivia, Chile; 4 Present Address: Production Unity of Advanced Therapy, Fundacion Ofalmologica de Santander, Clinica Carlos Ardila Lulle (FOSCAL Internacional), Bucaramanga, Colombia

**Keywords:** Mesenchymal stem cells, Multipotent stromal cells, Oral squamous cell carcinoma, Systemic administration, Cancer progression, Biosafety

## Abstract

**Background:**

Mesenchymal stem cells (MSCs) are a heterogeneous subset of stromal cells currently tested for multiple therapeutic purposes. Their potential to home into tumors, to secrete trophic/vasculogenic factors, and to suppress immune response raises questions regarding their biosafety. Our aim was to evaluate whether systemically administered allogeneic MSCs modify the natural progression of precancerous lesions and whether their putative effect depends on cancer stage and/or cell dose.

**Methods:**

Oral squamous cell carcinoma (OSCC) was induced in Syrian golden hamsters by topical application of 7,12-dimethylbenz[a]anthracene in one buccal pouch. At hyperplasia, dysplasia, or papilloma stage, animals received intracardially the vehicle or 0.7 × 10^6^, 7 × 10^6^, or 21 × 10^6^ allogeneic bone marrow-derived MSCs/kg. OSCC progression was assessed according to the presence of erythroplakia and leukoplakia, extent of inflammation and vascularization, and appearance, volume, and staging of tumors. Also, the homing of donor cells was studied.

**Results:**

Precancerous lesions progressed from hyperplasia to dysplasia in 2 weeks, from dysplasia to papilloma in 3 weeks, and from papilloma to carcinoma in 4 weeks. This time course was unmodified by the systemic administration of MSCs at hyperplasia or dysplasia stages. When MSCs were administered at papilloma stage, lesions did not progress to carcinoma stage. Tumors developed in hamsters receiving 0.7 × 10^6^ or 7 × 10^6^ MSCs/kg at hyperplasia stage were significantly smaller than those found in control animals (25 ± 4 or 23 ± 4 mm^3^ versus 72 ± 19 mm^3^, *p* < 0.05). Similar results were obtained when 0.7 × 10^6^, 7 × 10^6^, or 21 × 10^6^ MSCs/kg were administered at papilloma stage (44 ± 15, 28 ± 7, or 28 ± 5 mm^3^ versus 104 ± 26 mm^3^, *p* < 0.05). For dysplasia stage, only the lower concentration of MSCs reached statistical significance (21 ± 9 mm^3^ versus 94 ± 39 mm^3^, *p* < 0.05). Animals receiving 21 × 10^6^ MSCs/kg at hyperplasia stage developed tumors larger than those found in animals that received the vehicle (147 ± 47 mm^3^ versus 72 ± 19 mm^3^, *p* < 0.05). Donor cells were rarely found in precancerous lesions.

**Conclusions:**

Systemically administered allogeneic MSCs do not aggravate the progression of precancerous lesions. Moreover, they preclude cancer progression and tumor growth.

**Electronic supplementary material:**

The online version of this article (10.1186/s13287-018-0878-1) contains supplementary material, which is available to authorized users.

## Background

Mesenchymal stem cells (MSCs) are self-renewable undifferentiated cells found in almost all adult tissues [1]. MSCs can be procured from living donors, can be efficiently expanded ex vivo [2], are hypoimmunogenic so a conditioning regimen is not required before their transplantation [3], home into injured tissues and tumors [4, 5], differentiate into tissue cells [6], secrete trophic factors [7], promote neovascularization [8], reduce oxidative stress [9], and modulate the immune response [10]. Thus, MSCs appear to be an ideal tool for cell-based therapies.

The number of people receiving MSCs worldwide is increasing [11]. Some of them are patients enrolled in clinical trials but many of them receive the cells in the context of commercial unproved medical or cosmetic offers. Results of clinical trials are promising, nonetheless safety issues regarding MSC-based therapies are still unproven [12].

Currently, the envisioned adverse effects related to MSC administration are embolization, acute or chronic rejection, zoonosis when animal-derived products are used for cell expansion, increased susceptibility to infections, or upcoming neoplasia [13, 14]. A systematic review of 36 clinical trials showed no association between the intravascular administration of MSCs and acute toxicity, organ complications, infections, or de-novo malignancies [15]. Paradoxically, in animal models it has been shown that MSCs have both antitumor and protumor effects [14, 16]. Discrepancies have been explained by the source, dose, or delivery route, and also by the type and stage of cancer.

In order to fulfill a conclusive safety profile of MSCs, large-scale controlled studies with longer follow-up of adverse events are indispensable. Together, novel biosafety concerns should be studied systematically. One of these is the impact of MSC administration on the progression of precancerous lesions. These lesions are underdiagnosed and precede skin, oral cavity, esophagus, lung, pancreas, bladder, gallbladder, breast, ovary, cervix, and prostate cancers [17].

The aim of our work was to evaluate whether systemically administered allogeneic MSCs modify the natural progression of precancerous lesions and whether their putative effect depends on cancer stage and/or cell dose. For this, we used a preclinical model of oral squamous cell carcinoma (OSCC) that reproduces the etiology, malignant features, and dynamics of human oral epithelial tumors [18, 19].

## Methods

### Study design

**Fig. 1:**
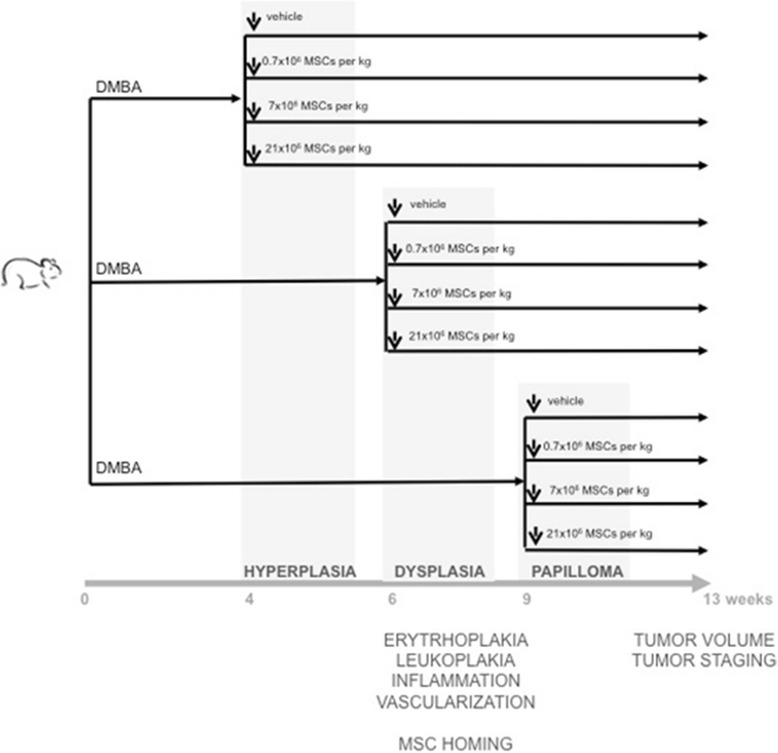
Study design. Syrian golden hamsters, male, 8 weeks old, were exposed to 7,12-dimethylbenz[a]anthracene (DMBA) three times a week. At hyperplasia, dysplasia, or papilloma stage, animals were randomized among experimental groups and received intracardially vehicle or 0.7 × 10^6^, 7 × 10^6^, or 21 × 10^6^ mesenchymal stem cells (MSCs)/kg. Every week, buccal pouches were assessed macroscopically. At the end of study period (13 weeks), tumors were analyzed microscopically. For each stage, homing of donor cells into precancerous lesions was evaluated 1 day after MSC administration

OSCC was induced in one buccal pouch of adult male hamsters. At hyperplasia, dysplasia, or papilloma stage, animals received intracardially the vehicle or 0.7 × 10^6^, 7 × 10^6^, or 21 × 10^6^ allogeneic bone marrow-derived MSCs/kg (Fig. 1). Every week, buccal pouches were assessed macroscopically. At the end of the study period (13 weeks), tumors were analyzed microscopically. For each stage, the homing of donor cells into precancerous lesions was evaluated 1 day after MSC administration.

The protocol was approved by the Ethic Committee of Facultad de Medicina Clinica Alemana-Universidad del Desarrollo (approval ID: 2011-14).

### Animals

A total of 270 Syrian golden hamsters (*Mesocricetus auratus*) were used in this study: 90 served as MSC donors and 180 were OSCC induced (60 hyperplasia, 60 dysplasia, 60 papilloma). Animals were housed at 22 °C, constant humidity, with a 12 h:12 h light–dark cycle, and food and water ad libitum. Hamsters were anesthetized by intraperitoneal injection of 20 mg/kg xylazine (Centrovet, Chile) and 200 mg/kg ketamine (Ilium, Argentina), and were euthanized by intraperitoneal injection of 40 mg/kg xylazine and 400 mg/kg ketamine. To regulate respiratory frequency during anesthesia, animals received sublingually 0.2 mg Viviram-V (Holliday Scott, Mexico).

### Induction of OSCC

Healthy male hamsters, 8 weeks old, weighing 142 ± 17 g, were painted three times a week in their right buccal pouch with a No. 4 camel-hair brush soaked with 50 μl of 0.5% (w/v) dimethylbenz[a]anthracene (DMBA) (Sigma-Aldrich, St. Louis, MO, USA) dissolved in mineral oil (Sigma-Aldrich) [18].

### Isolation, ex-vivo expansion, and characterization of MSCs

Healthy female hamsters, 8 weeks old, were euthanized and femurs and tibias were procured under sterile conditions. The epiphyses were removed and bone marrow cells were collected by flushing bones with sterile phosphate buffer saline (Gibco, Auckland, New Zealand). Recovered cells were resuspended in alpha-MEM (Gibco) supplemented with 10% fetal bovine serum (Gibco) and 80 μg/ml gentamycin (Sanderson Laboratory, Chile), and plated at a density of 0.25 × 10^6^ nucleated cells/cm^2^ in plastic tissue-culture dishes. After 72 h, nonadherent cells were removed by medium change. When foci reached confluence, adherent cells were detached with 0.25% trypsin, 2.65 mM EDTA (Gibco) and subcultured. At 70–80% confluence, cells were tripsinized, centrifuged, resuspended in alpha-MEM, counted, characterized, and injected.

Cell immunophenotyping was performed by flow cytometry after immunostaining with APC-conjugated anti-CD45 (BD Pharmingen, USA), FITC-conjugated anti-alpha smooth muscle actin (Sigma), and FITC-conjugated mouse anti-vimentin (Oncogen, USA) [2]. To assess differentiation potentials, cells were incubated with adipogenic or osteogenic induction media [20]. Seven and 14 days later, samples were stained with Oil Red O or Alizarin Red (Sigma) (Additional file 1: Figure S1).

### Systemic administration of MSCs

When the target OSCC stage was reached, animals were distributed randomly among the experimental groups. After anesthesia, the heart was palpated and the needle of a Tuberculin syringe (Terumo, Japan) was inserted at sternum height [21]. Once retrograde flow was observed, 600 μl of 5% hamster plasma in physiological serum (vehicle) or 0.1 × 10^6^, 1 × 10^6^, or 3 × 10^6^ MSCs resuspended in the vehicle were injected. These numbers of cells results in doses of 0.7 × 10^6^, 7 × 10^6^, or 21 × 10^6^ MSCs/kg, respectively.

### Macroscopic assessment of OSCC stages

Four, 6, 9, and 13 weeks after the first DMBA exposure, animals were anesthetized and the right buccal pouch was exposed and photographed using a digital camera (FUJIFILM-Finepi HS20 EXR or Olympus SP5 10). Two independent observers analyzed the photographs and classified lesions according to the absence/presence of erythroplakia, leukoplakia, inflammation, vascularization, ulcers, or exophytic nodules [18]. Tumors were measured with a digital caliper (Mitutoyo Sul Americana, Brazil). The tumor volume was calculated using the formula: tumor volume (mm^3^) = 0.52 × width (mm)^2^ × length (mm) [22].

### Microscopic assessment of OSCC stages

Thirteen weeks after the first DMBA exposure, animals were euthanized, right buccal pouches were procured, and tumors were resected. Samples were fixed in 10% buffered formalin (Merck, USA), embedded in paraffin (Merck), and sectioned. Tissue sections of 4 μm were deparaffinized with Neoclear (Merck), rehydrated with graded alcohols, stained with hematoxylin–eosin (Merck), and visualized with a light microscope (DM2000; Leica, Germany). Images were captured with a digital camera (DFC295; Leica). Samples were classified as hyperplasia, dysplasia, papilloma, or carcinoma as described previously [18, 19].

Histological analyses were performed blind by three independent observers; one of them is a pathologist expert in oral diseases.

### Detection of donor MSCs in recipient mucosa

MSCs were labeled with 25 μM CellTracker Red CMTPX (Invitrogen, USA) for 20 min at 37 °C. After washing, cells were tripsinized and injected intracardially (see earlier). The day after, hamsters were euthanized, and buccal pouches were procured and fixed with OCT (Tissue-Teck, USA). Cryosections of 10 μm thickness were stained with DAPI (Invitrogen, USA), mounted with epifluorescence medium (Dako, USA), and visualized with an epifluorescence microscope (DM2000 (Leica) or Flv10i.doc (Olympus, USA)) [6].

### Statistical analysis

As data distribution was nonparametric, results were presented as median ± SEM. Tumor volume comparisons were performed using one-way ANOVA test followed by Dunn’s post test. Statistical analyses were performed using StatGraph Prism 5.0 software. *p* < 0.05 with a confidence interval of 95% was considered statistically significant.

## Results

### Systemically administered allogeneic MSCs do not accelerate the progression of precancerous lesions

**Fig. 2:**
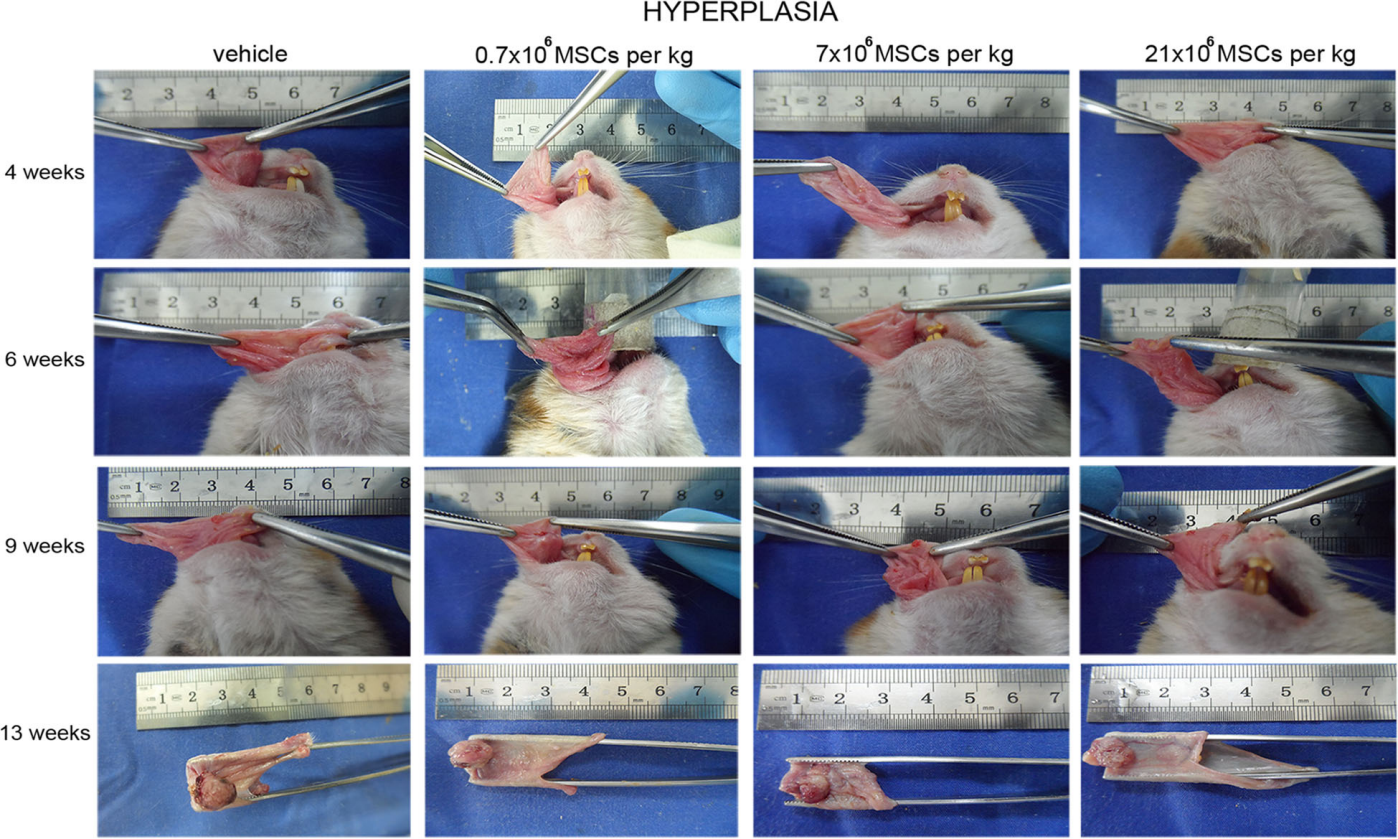
Systemically administered allogeneic MSCs do not accelerate progression of precancerous lesions. At hyperplasia stage, hamsters received intracardially vehicle or 0.7 × 10^6^, 7 × 10^6^, or 21 × 10^6^ MSCs/kg. Progression of precancerous lesions was assessed macroscopically (*n* = 14). MSC mesenchymal stem cell

**Fig. 3:**
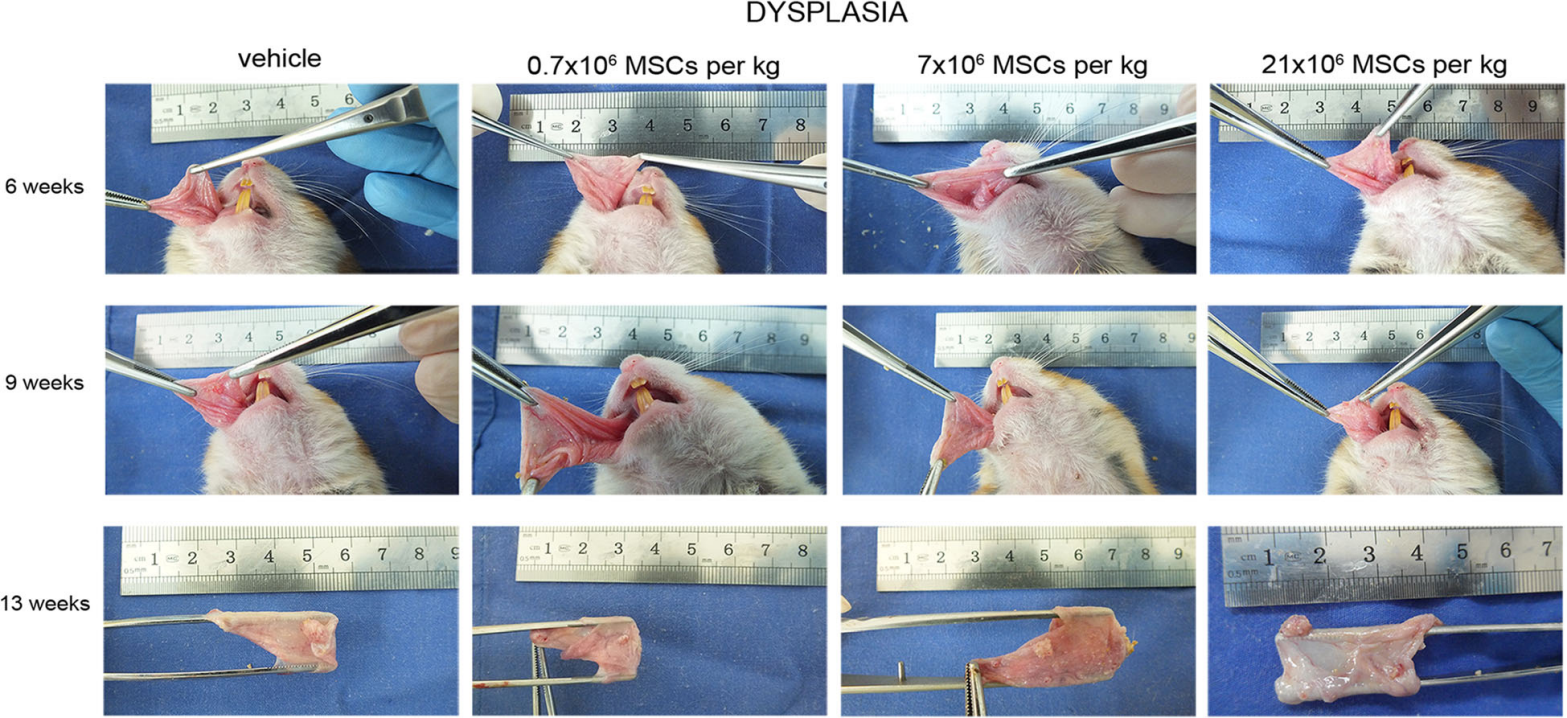
Systemically administered allogeneic MSCs do not accelerate progression of precancerous lesions. At dysplasia stage, hamsters received intracardially vehicle or 0.7 × 10^6^, 7 × 10^6^, or 21 × 10^6^ MSCs/kg. Progression of precancerous lesions was assessed macroscopically (*n* = 14). MSC mesenchymal stem cell

**Fig. 4:**
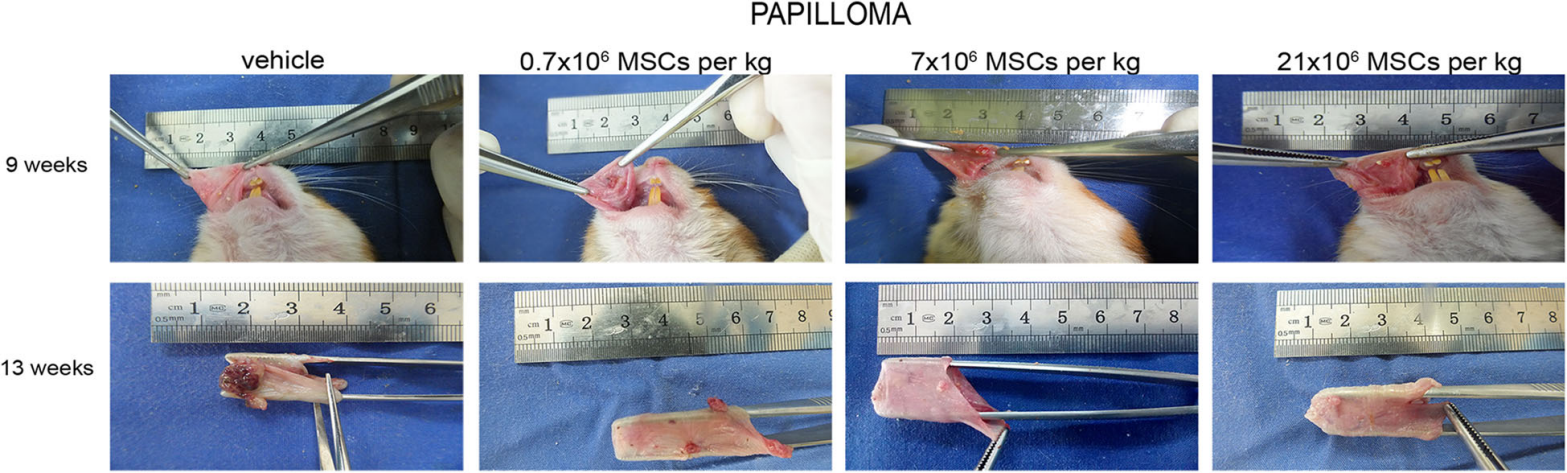
Systemically administered allogeneic MSCs do not accelerate progression of precancerous lesions. At papilloma stage, hamsters received intracardially vehicle or 0.7 × 10^6^, 7 × 10^6^, or 21 × 10^6^ MSCs/kg. Progression of precancerous lesions was assessed macroscopically (*n* = 14). MSC mesenchymal stem cell

Four weeks after the first DMBA exposure, buccal pouches of animals that received the vehicle were retracted, and erythroplakia, leukoplakia, or both were evident (Fig. 2). Together, an engrossment of the mucosa due to inflammation was observed. Two weeks later (6 weeks after the first DMBA exposure), mucosa engrossed and lost elasticity; no tumors were visible at this time point. Three weeks later (9 weeks after the first DMBA exposure), exophytic lesions protruded from the surface and therefore tumors were measurable. Four weeks later (13 weeks after the first DMBA exposure), buccal pouches retracted and stiffened, presenting features of necrosis and larger tumors. This time course was unchanged in animals receiving MSCs at hyperplasia (Fig. 2) or dysplasia (Fig. 3) stage. At week 13, the buccal pouches of animals receiving MSCs at papilloma stage neither retracted nor presented features of necrosis (Fig. 4). Also, tumors did not enlarge.

### Systemically administered allogeneic MSCs do not increase tumor malignancy

**Fig. 5:**
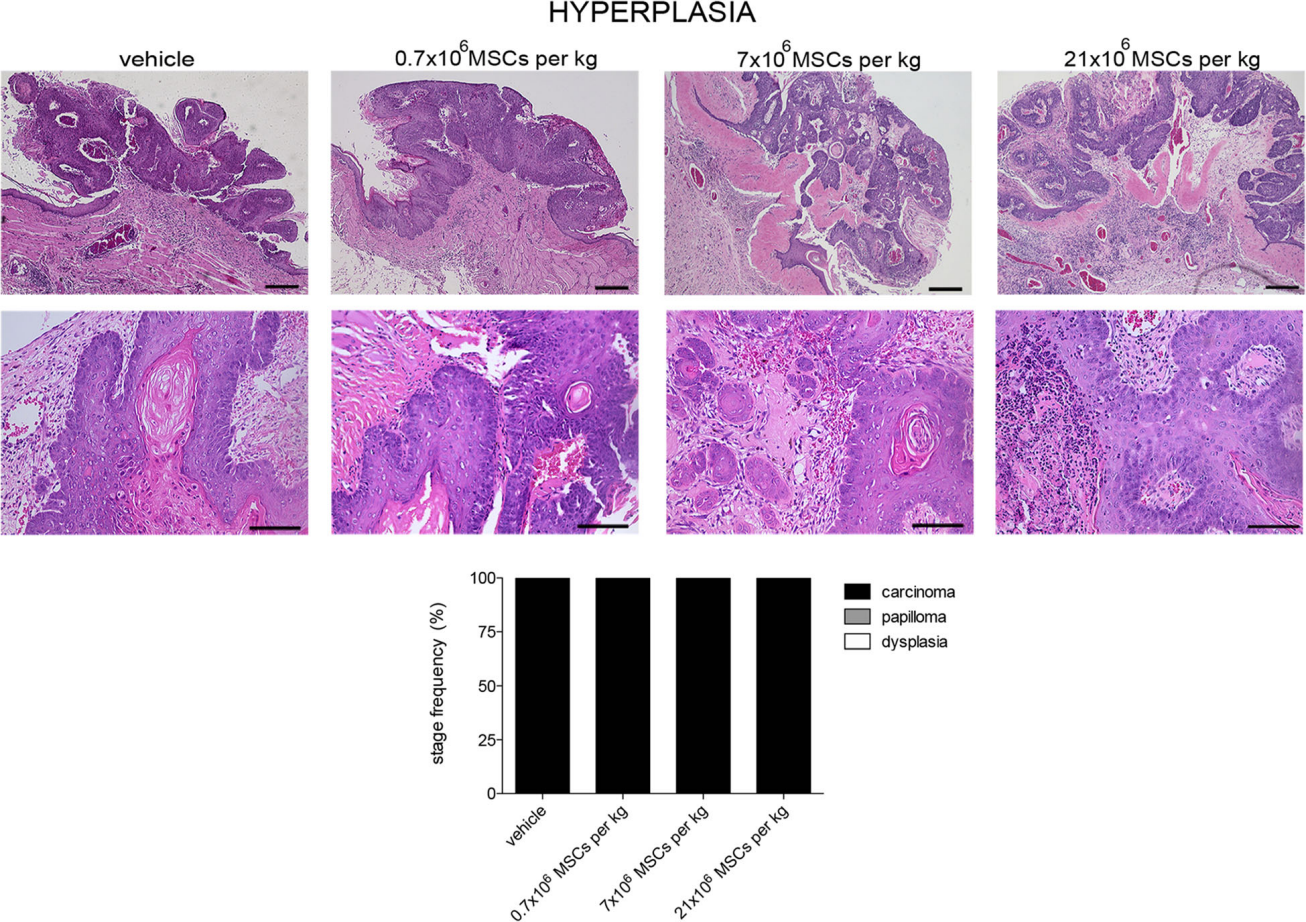
Systemically administered allogeneic MSCs do not increase tumor malignancy. At hyperplasia stage, hamsters received intracardially vehicle or 0.7 × 10^6^, 7 × 10^6^, or 21 × 10^6^ MSCs/kg. At end of study period (13 weeks), tumors were assessed microscopically. Stage frequency was calculated for each group. Bar = 100 μm (number of replicates = 6, *n* = 14). MSC mesenchymal stem cell

**Fig. 6:**
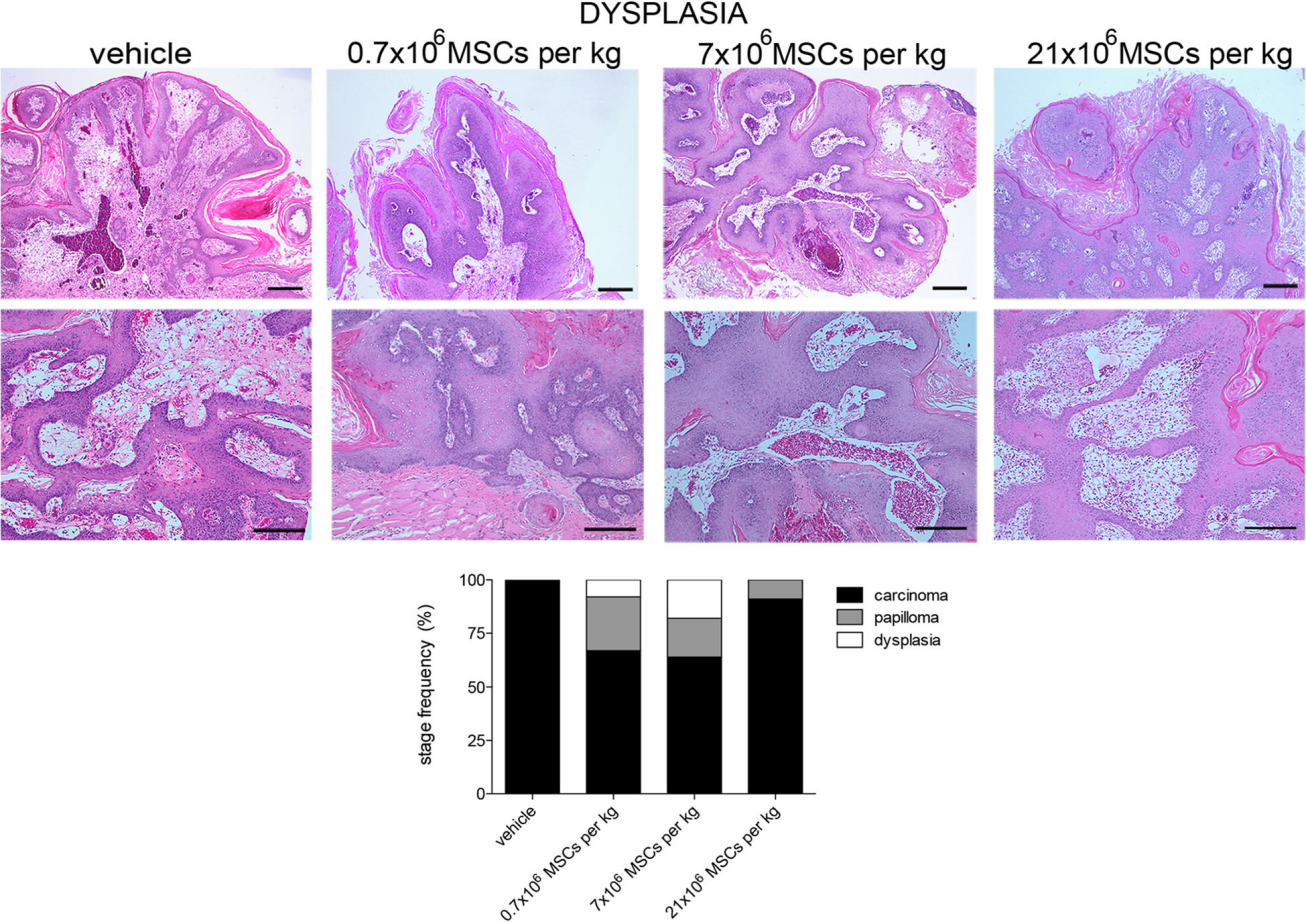
Systemically administered allogeneic MSCs do not increase tumor malignancy. At dysplasia stage, hamsters received intracardially vehicle or 0.7 × 10^6^, 7 × 10^6^, or 21 × 10^6^ MSCs/kg. At end of study period (13 weeks), tumors were assessed microscopically. Stage frequency was calculated for each group. Bar = 100 μm (number of replicates = 6, *n* = 14). MSC mesenchymal stem cell

**Fig. 7:**
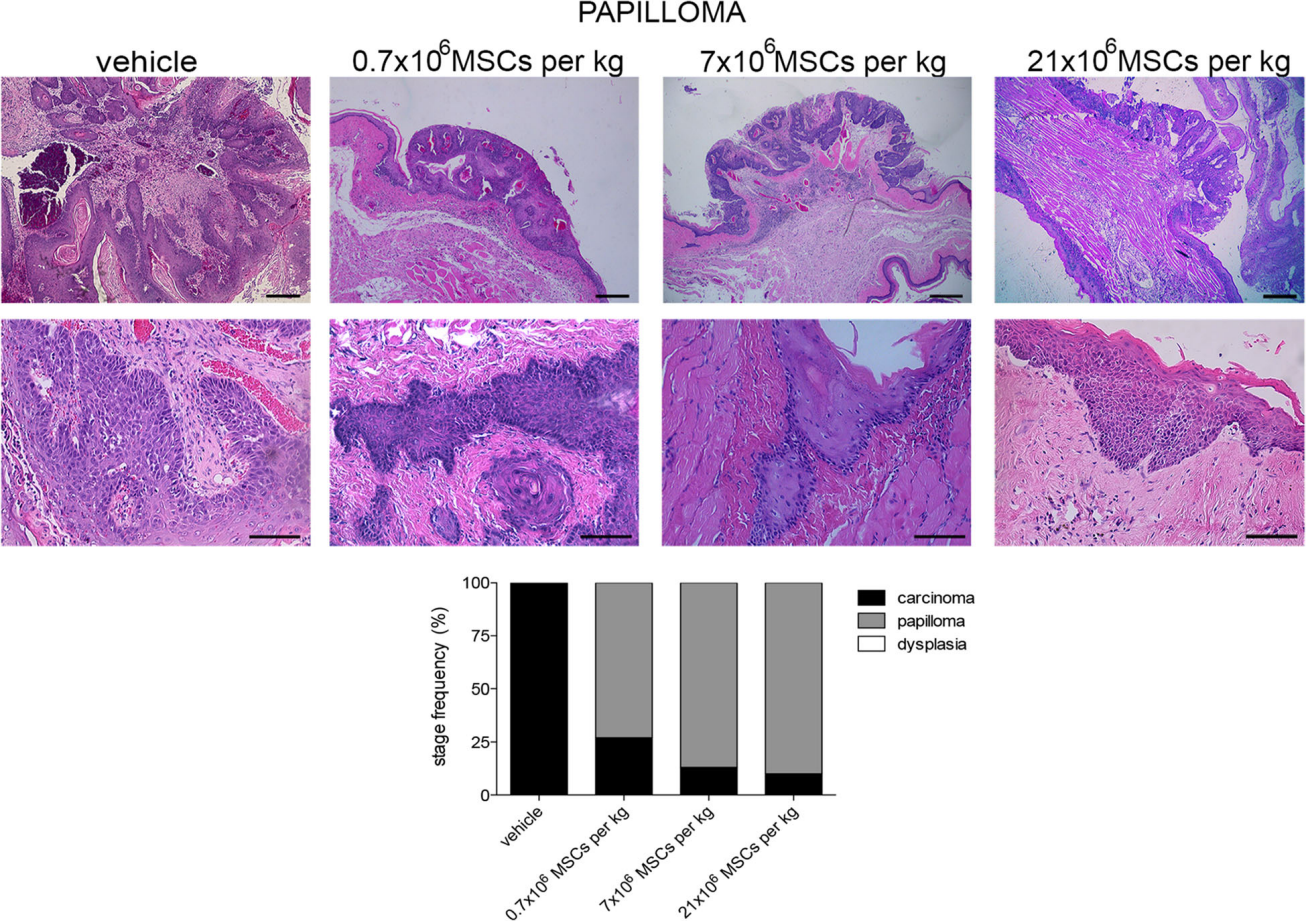
Systemically administered allogeneic MSCs do not increase tumor malignancy. At papilloma stage, hamsters received intracardially vehicle or 0.7 × 10^6^, 7 × 10^6^, or 21 × 10^6^ MSCs/kg. At end of study period (13 weeks), tumors were assessed microscopically. Stage frequency was calculated for each group. Bar = 100 μm (number of replicates = 6, *n* = 14). MSC mesenchymal stem cell

Thirteen weeks after the first DMBA exposure, the oral mucosa of animals receiving the vehicle showed cell and nuclear pleomorphism, the epithelium stratification was lost, the basal membrane appeared discontinuous, vessels were aberrant (large size and discontinuous wall), and inflammatory foci were abundant (Figs. 5, 6, and 7). Therefore, at the end of the study period all animals receiving the vehicle presented carcinoma. The same was observed in hamsters receiving MSCs at hyperplasia stage (Fig. 5). For animals that received 0.7 × 10^6^, 7 × 10^6^, or 21 × 10^6^ MSCs/kg at dysplasia stage, the frequencies of lesions that progressed to carcinoma were 67, 66, and 90%, respectively (Fig. 6). When MSC administration was performed at papilloma stage, the frequencies of malignant transformation were 27, 13, and 10% for 0.7 × 10^6^, 7 × 10^6^, or 21 × 10^6^ MSCs/kg, respectively (Fig. 7). Most of the tumors found in animals receiving MSCs at papilloma stage remained at this stage, and showed continuous basal membrane and rare epithelial cells invading the stroma.

### Low doses of systemically administered allogeneic MSCs preclude OSCC tumor growth

**Fig. 8:**
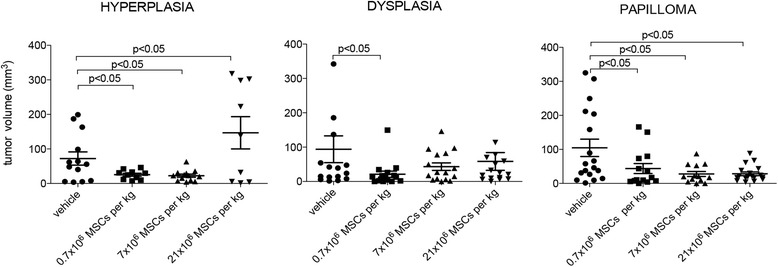
Low doses of systemically administered allogeneic MSCs preclude OSCC tumor growth**.** At hyperplasia, dysplasia, or papilloma stage, hamsters received intracardially vehicle or 0.7 × 10^6^, 7 × 10^6^, or 21 × 10^6^ MSCs/kg. At end of study period (13 weeks), tumor volume was assessed. (number of replicates = 2, *n* = 14). MSC mesenchymal stem cell

Thirteen weeks after the first DMBA exposure, the mean size of tumors developed in animals receiving the vehicle at hyperplasia, dysplasia, or papilloma stage was 90 ± 28 mm^3^ (72 ± 19 mm, 94 ± 39 mm, and 104 ± 26 mm, respectively) (Fig. 8). The administration of 0.7 × 10^6^ or 7 × 10^6^ MSCs/kg at hyperplasia stage resulted in tumors three times smaller (25 ± 4 mm^3^ or 23 ± 4 mm^3^). However, the high dose of MSCs resulted in tumors two times bigger (147 ± 47 mm^3^). All of the observed differences were statistically significant (*p* < 0.05) (Fig. 8). When MSCs were administered at dysplasia stage, no difference was observed when compared with the control, except for the low dose (21 ± 9 mm^3^; *p* < 0.05). Animals that received MSCs at papilloma stage developed tumors two to three times smaller than those presented in control animals (0.7 × 10^6^ MSCs/kg, 44 ± 15 mm^3^; 7 × 10^6^ MSCs/kg, 28 ± 7 mm^3^; 21 × 10^6^ MSCs/kg, 28 ± 5 mm^3^; *p* < 0.05 for the three comparisons).

### Systemically administered allogeneic MSCs rarely home into precancerous lesions

**Fig. 9:**
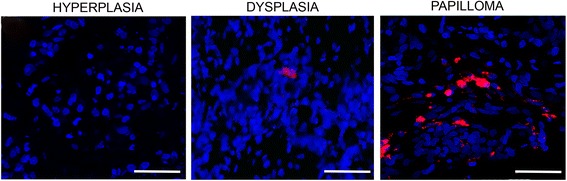
Systemically administered allogeneic MSCs rarely home into precancerous lesions. At hyperplasia, dysplasia, or papilloma stage, hamsters received intracardially 21 × 10^6^ CMTPX-labeled MSCs/kg. One day later, donor cells were sought in precancerous lesions. Bar = 50 μm (number of replicates = 6, *n* = 3)

One day after intracardial administration, MSCs labeled with CMTPX (donor cells) were scarcely found in the oral mucosa of animals presenting precancerous lesions at dysplasia or papilloma stage, but not at hyperplasia stage (Fig. 9). Since not all of the sections analyzed presented donor cells and the number of donor was very low (1–3 cells/field), quantitative analysis was not performed.

## Discussion

In this study, we present evidence in support of the hypothesis that systemically administered allogeneic MSCs neither accelerate the progression nor increase tumor malignancy of precancerous lesions. Moreover, we show that depending on the stage of the precancerous lesion and the dose of cells, the systemic administration of allogeneic MSCs precludes OSCC progression and tumor growth. To our knowledge, these observations are without precedent since the antitumor effect already described for systemically administered MSCs has been proven in animal models bearing preestablished tumors or receiving tumor cells together with the MSCs [23, 24].

The fact that systemically administered allogeneic MSCs do not aggravate the progression of precancerous lesions is a relevant safety outcome. Due to its simplicity and the ability of MSCs to home into injured tissues, intravenous injection is the preferred route in currently proposed MSC-based therapies. Most frequent malignancies arise from precancerous lesions, which are asymptomatic and underdiagnosed [25, 26]. Thus, there is a high probability that candidate patients for MSC-based therapies carry precancerous lesions, unnoticed by themselves and by clinicians. Here, we provide empirical evidence that this would not be a major health risk.

Regarding the beneficial effects of the systemic administration of MSCs, we observed a deterrence of OSCC progression when cells were injected at dysplasia or papilloma stage but not at hyperplasia stage. Precancerous lesions at advanced stages presented chronic inflammation [27]. Thus, only at later stages might MSCs exert their immunomodulatory effect impairing cancer progression [28]. Since our study design lasted up to a fixed time point (13 weeks), we do not know whether this is a delay or suppression of OSCC progression. Prolonged follow-up is required to address this point.

The preclusion of OSCC tumor growth was observed irrespective of the stage of precancerous lesion but varied according to the number of cells injected. The dose-dependent effect of MSCs has also been proven in other sceneries. In an ovine model of myocardial infarction it has been shown that the low doses (25 × 10^6^ or 75 × 10^6^ MSCs) but not the high ones (225 × 10^6^ or 450 × 10^6^ MSCs) attenuated infarct expansion and preserved cardiac function [29]. When the safety and efficacy of 20 × 10^6^, 100 × 10^6^, or 200 × 10^6^ MSCs were tested in patients with ischemic cardiomyopathy, the greatest effect in functional capacity was reached with the low dose of cells [30]. The improvement of fistula healing was observed when 30 × 10^6^ MSCs were administered to patients with Crohn’s disease, but not when the dose was 90 × 10^6^ MSCs [31]. In all of these studies, the higher doses of MSCs were less effective or have no effect. In our study, the high dose of MSCs administered at hyperplasia stage resulted in an undesirable effect (bigger tumors). This could be explained by the fact that the administration of a large number of cells in a barely unhealthy animal might trigger an immune response against the donor cells, resulting in their deactivation or elimination, and/or might promote a long-term immunosuppression that facilitates posterior tumor growth [32].

As tumor growth restriction was not an expected outcome of the present work, our original study design did not consider exploration of the putative mechanisms behind it. Previously, we showed that local administration of allogeneic MSCs at dysplasia or papilloma stage precludes OSCC tumor growth (unpublished results, [19]). While at dysplasia stage MSCs impede mast cell infiltration and diminish tissue vascularization, at papilloma stage donor cells diminish proliferation, increase apoptosis, and reduce epithelial dedifferentiation. Thus, the cellular consequences of MSC administration vary according to the stage of precancerous lesion. Here, we observed that systemically administered allogeneic MSCs rarely home into precancerous lesions since we hypothesize that beneficial effects are due to systemic changes, as described for diseases like autoimmune encephalomyelitis or type 1 diabetes mellitus [10, 33].

Although the limitation of our work is that results were obtained in an animal model for one type of cancer, the study represents a new biosafety insight for MSC-based cell therapy. Also, it is a first approach to the use of MSCs for the prevention of OSCC.

## Conclusions

Systemically administered allogeneic MSCs do not aggravate the progression of precancerous lesions. Moreover, they preclude cancer progression and tumor growth.

## Additional file

Additional file 1: **Figure S1.** Characterization of MSCs isolated from bone marrow of Syrian golden hamster. Immunophenotype (**a**) and differentiation potential (**b**). Dashed line, mean fluorescence intensity of isotype control. Bar = 100 μm (*n* = 4). (TIF 1623 kb)

## Abbreviations

ANOVA: Analysis of variance
APC: Allophycocyanin
CMTPX: 5-({[4-(Chloromethyl)phenyl]carbonyl}amino)-2-(1,2,2,4,8,10,10,11-octamethyl-10,11-dihydro-2H-pyrano[3,2-g:5,6-g']diquinolin-1-ium-6-yl)benzoate
DAPI: 4′,6-Diamidino-2-phenylindole dihydrochloride
DMBA: 7,12-Dimethylbenz[a]anthracene
EDTA: Ethylenediaminetetraacetic acid
FITC: Fluorescein isothiocyanate
MEM: Minimum essential medium
MSC: Mesenchymal stem cell
OCT: Optimum cutting temperature embedding material
OSCC: Oral squamous cell carcinoma
SEM: Standard error of the mean
