# Supplementary figures and images for: Systemically administered allogeneic mesenchymal stem cells do not aggravate the progression of precancerous lesions: a new biosafety insight
Source: Stem Cell Res Ther. 2018 May 11;9:137. doi: 10.1186/s13287-018-0878-1 (PMC5948822; doi:10.1186/s13287-018-0878-1)

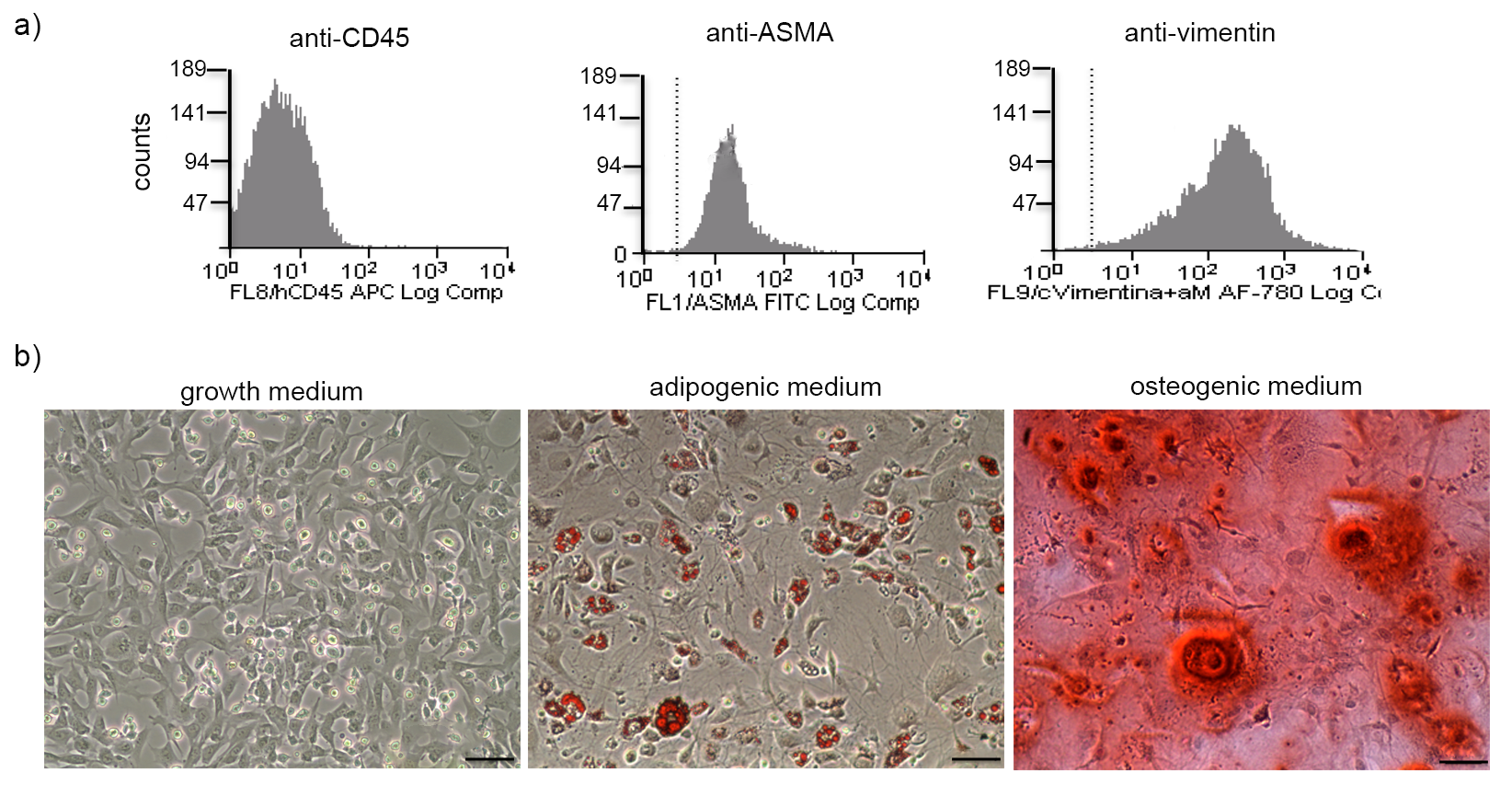

Supplement: Supplementary file 1 — Figure S1. Characterization of MSCs isolated from bone marrow of Syrian golden hamster. Immunophenotype (a) and differentiation potential (b). Dashed line, mean fluorescence intensity of isotype control. Bar = 100 μm (n = 4). (TIF 1623 kb) [file 13287_2018_878_MOESM1_ESM.tif]
